# Why did the pheasant cross the road? Long-term road mortality patterns in relation to management changes

**DOI:** 10.1098/rsos.170617

**Published:** 2017-10-04

**Authors:** Joah R. Madden, Sarah E. Perkins

**Affiliations:** 1 Centre for Research in Animal Behaviour, Psychology, University of Exeter, Exeter EX4 4QG, UK; 2 School of Biosciences, Cardiff University, Museum Avenue, Cardiff CF10 3AX, UK

**Keywords:** game management, *Phasianus colchicus*, roadkill, traffic collisions

## Abstract

Pheasants (*Phasianus colchicus*) are commonly killed on UK roads, presenting a threat to motorists and a loss to the game shooting industry. Pheasants may be inherently susceptible, or the recent increase in their artificial rearing and release may have exacerbated the situation, either through population increases or because artificial rearing has altered movement behaviour. We compared intra-annual patterns of roadkill reported in the UK from the 1960s (prior to the onset of mass release programmes) with that from the 2010s (when pheasant release was well established and widespread), considering roadkill sex and locations and accounting for changes in traffic levels. Pheasants in the UK are disproportionately likely to be reported killed on roads. However, this likelihood has not changed notably over the past 50 years. Instead, the timing of roadkill has changed. Pheasants in the 2010s are no longer susceptible during their breeding season, unlike in the 1960s, perhaps because relatively few breed successfully. Instead, roadkill first peaks in September–November as pheasants disperse from release pens, females first. Roadkill declines over winter, but when supplementary feeding ceases in February, we see a second peak in roadkill. Roadkill rates are higher in regions of the UK where there is little arable farming and hence natural food supplies are scarce.

## 1. Introduction

A pheasant (*Phasianus colchicus*) lying dead on the side of the road is a common sight in the UK. Collisions with pheasants were implicated in 65 accidents per year (between 1999 and 2003) that led to human injury, with approximately 6% of these leading to serious injury or death of humans [1]. Pheasants may be commonly killed on the roads simply because there are large numbers (approx. 35 million [2]) of them released in the UK for shooting each year. The scale of pheasant release has increased by approximately 900% since the 1960s as efficient artificial rearing methods have been developed [3]. In general, species of birds more abundant next to roads are more likely to be involved in vehicle collisions [4,5]. Alternatively, pheasants as a species may exhibit traits that make them especially susceptible to vehicle collisions because they are omnivorous [6], have short flight distances [5], and have relatively small brains [7]. Finally, pheasants in the UK may be unusually vulnerable to vehicle collision because of the conditions under which the vast majority of birds in the UK have been reared and managed post-release. Most pheasants in the UK have been mechanically incubated and reared in the absence of adults in large groups of several hundred same-age peers for five to eight weeks prior to release [8]. Pheasants artificially reared in barren environments had poorer spatial memory [9]; reared pheasants are typically poorer flyers than birds born in the wild [10]; and released birds become dependent on supplementary feeding at fixed points during the shooting season, after which feeding is commonly stopped and pheasants have to search for novel feeding sites during late winter/early spring [11].

Pheasants are large (approx. 1.4 kg♂, approx. 1 kg♀) birds and the males are visually conspicuous [8]. Consequently, they are commonly and reliably recorded in records of roadkill, making up 2–29% of animals reported killed on the roads in the UK, Europe and the USA [12–14]. This allows us to test whether pheasants are being killed at a rate proportionate to their abundance, or whether they are disproportionately killed, perhaps because of their natural physiology and/or behaviour. Recording carcasses by the roadside risks underestimating actual roadkill mortality levels [15], but by comparing proportion data we can account for some of these factors such as differential decay, detectability and scavenging rates. Records of roadkill have been collected since the 1940s [16], and this allows an analysis of how patterns of roadkill have changed over that time. We compared patterns of pheasant mortality in the UK from the 1960s, when most of this species in the UK were hatched and raised in the wild under natural conditions, with patterns from the 2010s when most UK pheasants are hatched in incubators and reared under intensive, artificial, conditions. This permits us to ask whether the change in the origin of pheasants, from predominantly wild-born to predominantly artificially reared has affected their patterns of roadkill in terms of their susceptibility to vehicle collisions and/or the periods when they are most vulnerable to roadkill.

Changes in patterns of mortality could be driven by changes in motoring behaviour or roadkill reporting methods. Roadkill rates alter with traffic volume and speed [12], both of which have increased since the 1960s. Therefore, it is necessary to account for the confounding factors by comparing changes in the mortality patterns of other bird species over the same time period. We used a second bird, the pigeon, which is commonly killed on roads but has not had the same dramatic change in life history in terms of switching from natural breeding to being reared and released over the past 50 years as our comparator species.

Studies of roadkill commonly trade-off precision in the exact sites of death for an increase in sample sizes. For example, a detailed study of a single 3.9 km road stretch yielded data on the exact locations of death, but was restricted to only 56 individual birds [13]. By contrast, an American study recorded 24 244 roadkill birds, but could only locate them on particular Interstate Highways to a precision of many tens of kilometres [12]. A more fine-scale resolution of roadkill sites, especially when related to surrounding environmental conditions, would permit a better understanding of factors causing roadkill. In the case of pheasants, this could include the (lack of) availability of food in the surrounding area which may prompt movement across roads or risky foraging behaviour. A better understanding of factors that lead to roadkill of pheasants would inform game management practices that could be implemented in order to reduce incidents of roadkill, and consequent human injuries and fatalities.

## 2. Material and methods

### 2.1. Data sources

Data for pheasant mortality in the UK during a 4-year period of intensive pheasant release and management (January 2013–December 2016) were obtained as part of ‘Project Splatter’ (https://projectsplatter.co.uk/). This citizen science project involved members of the public reporting *ad hoc* sightings of roadkill wildlife detected across the UK via a variety of methods (social media, email and app) to a central database [17]. Sightings were date stamped and could, but did not always, include the location, sex and age of the bird. We specifically extracted numbers of pheasants, pigeons (as a control species being the next most common bird reported killed, with reports of wood pigeons, *Columba palumbus*, and feral pigeons, *C. livia*, combined due to difficulty distinguishing these two species) and other birds of all species reported killed each month. This comprised 2990 pheasants and 1554 pigeons as part of 7849 records of birds killed (see the electronic supplementary material for full data). Data for pheasant mortality in the UK during a period when relatively few pheasants were artificially reared and released and their subsequent management was less intensive were obtained from three published studies, also spanning 4 years (1957–1961) covering both surveys and more detailed regional surveys for which monthly numbers of pheasants and numbers of all birds killed in vehicle collisions were reported [14,18,19]. This comprised 266 pheasants as part of 3932 records of birds killed. The raw monthly data are presented in the electronic supplementary material.

### 2.2. Interpreting vehicle collision data

Surveying bird mortality due to vehicle collisions is imprecise and subject to multiple biases. The actual death rates may be 12–16 times the number of corpses found due to rapid scavenging [15]. Recording rates may vary with body size, both in terms of visibility and likelihood of being removed by scavengers. Between 60 and 97% of carcasses disappear within the first 36 h of being on roads [20]. Where the roadkill is a small animal, rates may be as high as 89% removal within the first 24 h [21]. Observations on larger vertebrates find 40% disappear within 7 days [22]. Death rates may vary with road surface [23], traffic density [24] and speeds [25] and the habitats bordering the road [26,27]. There have not been consistent, long-term surveys of bird traffic fatalities in the UK, but rather there have been a series of independent surveys that have all used different survey intensities, methodologies and study areas. These range from diligent authors cycling set sort routes on a weekly basis, stopping to investigate and identify every animal encountered (e.g. [13]), to citizen science surveys in which no attempt is made to standardize survey effort or observer bias in the collection of data (e.g. Project Splatter [17]). Therefore, data from multiple surveys must be standardized to permit comparisons to be made.

We were interested in two facets of mortality. First, whether there were population-level patterns in pheasant deaths across any given year. For this, it was necessary to correct for both sampling effort and traffic pressure. For example, people may be less likely to drive during winter and those that do may be less able to report roadkill, because it is harder to see in the dark. Additionally, because of the longer nights, the risk to diurnal birds is reduced in winter as they spend less time on roads. Therefore, we calculated the proportion of birds reported as roadkill which were identified as pheasants. This measure also permitted us to compare relative rates of roadkill across long time periods to ask whether pheasants were becoming proportionately more likely to be killed compared with other bird species. However, this approach is susceptible to distortion by the patterns of behaviour and mortality of other species. For example, a relative decrease in pheasant roadkill may be due to a rise in the roadkill of another species, rather than reflecting a real decline in pheasant deaths, or if comparisons are made across time periods it may be distorted by changes in the size of the pheasant population, which we know to have increased substantially from the 1960s to the 2010s [3,28]. Therefore, we used a second measure of the proportion of roadkill pheasants in a year that were killed in a particular month. This measure allowed us to control for variations in the population sizes and mortality patterns of non-pheasant birds and so look at patterns of mortality within the year. However, this approach is more susceptible to biases caused by sampling effort and traffic pressure and so has to be considered alongside our previous measure. We deployed these two methods to explore patterns in roadkill across the year, between the two time periods (1960s versus 2010s) and across different regions of the UK within a time period. Finally, we looked at whether, within pheasants, the sexes were differentially reported as roadkill by first considering the proportion of all roadkill pheasants per month that were males or females (these were not inversely linked to one another because for many reports, no sex was given), and second by considering the relative proportion of sexed birds that were females.

### 2.3. Regional differences in arable farming

We calculated the percentage of the total farmed area that was arable crops or bare fallow ground for each of the nine regions of England as reported by DEFRA in 2016 [29]. Two regions (East Midlands and Eastern) had greater than 60% of farmed land as arable, whereas the other seven regions were all less than 50% arable with a mean of 34%. Therefore, we classed any pheasants (*n* = 986) reported from Eastern and East Midlands as coming from areas of high arable farming, with other pheasants reported from England (*n* = 1650) as coming from areas of low arable farming. We excluded records from Wales, Scotland and Northern Ireland (*n* = 363) because the farming data were not collated in the same way.

### 2.4. Statistical analysis

We used generalized linear models with a binomial function and logit link to test whether the patterns of mortality, given by the numbers of roadkill pheasants reported per month as the numerator and either the total number of roadkill birds in that month or the total number of roadkill pheasants in the year/period of survey as the denominators. We included year and month as fixed factors and the interaction between them to explicitly test whether patterns of mortality differed across the 4 years of the data collected via Project Splatter. Using the 2010 data, we ran two separate models to ask whether region or sex was related to patterns of roadkill. In each case, we combined data across the 4 years and included month and either sex or region and their interactions. We did not include both factors in a single model because only 1197/2990 (40%) of records identified the bird's sex and we would have lost a large amount of data by excluding the other 60% of records in our analysis of regional patterns. All analyses were conducted using SPSS v23.

## 3. Results

### 3.1. Differences in levels of mortality before and after the introduction of large-scale pheasant releases

Between 1960 and 1961, pheasants comprised 6.8% of roadkill birds and their breeding population was estimated at 200 000–1 million, comprising approximately 0.1–0.5% of the UK breeding bird population of 210 million [30]. Therefore, prior to the introduction of large-scale pheasant releases, the proportion of pheasants reported killed by vehicle collisions was approximately 13 times greater than that expected given their breeding population size.

Between 2013 and 2016, pheasants comprised 38.1% of reported roadkill birds and their breeding population (in 2011) was estimated at approximately 5.6 million, comprising approximately 3.2% of the UK bird population of 166 million birds [31]. When we considered biomass, pheasants (and other gamebirds) comprised 23.2% of the mass of the breeding population [31]. Following the breeding season, there are estimated to be approximately 400 million birds in the UK in autumn, including approximately 28 million pheasants (7% of the total) [28]. Therefore, following the introduction of large-scale pheasant releases, the proportion of roadkill pheasants was 11.9 times greater than that expected given their breeding population size, 5.44 times greater than their wintering population size and 1.6–1.3 times greater than expected given their body mass.

By contrast, between 1960 and 1961, pigeons comprised 2.4% of roadkill birds and their breeding population was estimated at approximately 8 million, comprising 3.8% of the UK breeding bird population of 210 million [30]. During the 1960s, the proportion of pigeons reported killed by vehicle collisions was approximately 63% of that expected given their breeding population size.

Between 2013 and 2016, pigeons comprised 19.8% of reported roadkill birds and their breeding population (in 2011) was estimated at approximately 7.2 million pairs (14.4 million birds), comprising approximately 8.7% of the UK bird population of 166 million birds [31]. Pigeons and doves comprised 22.5% of the mass of the breeding population [31]. Therefore, pigeons were about 2.27 times more likely to be killed on the road than expected given their breeding population and killed at about 88% of the level expected given their body mass.

**Figure 1.**
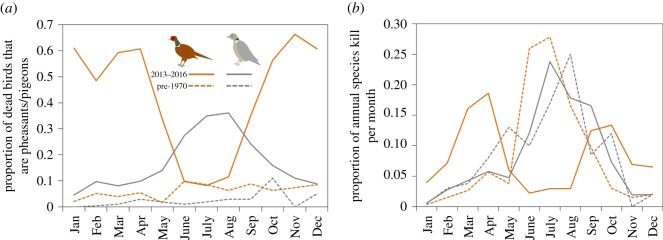
Pheasants (orange lines) and pigeons (grey lines) killed as (*a*) a proportion of total birds reported killed by vehicle collisions and (*b*) a proportion of each category killed per year in each month, for pheasants between 2013 and 2016 (solid orange line) and before 1970 (dashed orange line) and for pigeons between 2013 and 2016 (solid grey line) and before 1970 (dashed grey line).

Therefore, the proportion of roadkill pheasants reported compared to other birds was 5.6 times higher in the 2010s compared to the 1960s with a corresponding breeding population increase of 5.6 times. By contrast, the proportion of roadkill pigeons was 8.25 times higher in the 2010s compared to the 1960s, with a corresponding breeding population increase of 1.8 times (figure 1*a*).

### 3.2. Are patterns of mortality consistent across short spans of years?

**Figure 2.**
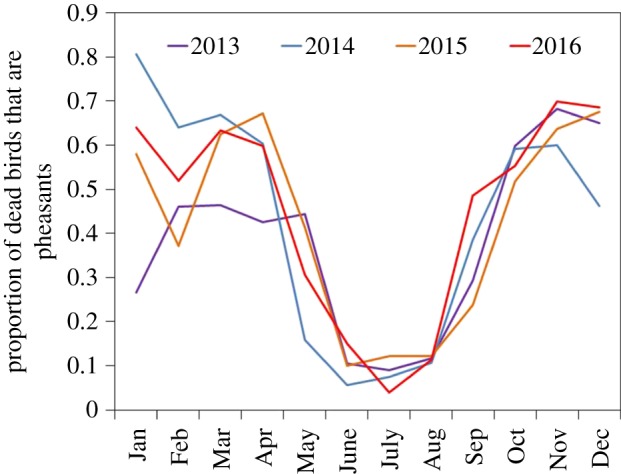
Pheasants reported killed in vehicle collisions each month as a proportion of the total number of birds reported to Project Splatter in that month across 4 years (2013–2016).

The general pattern of the number of roadkill pheasants as a proportion of all the birds reported to Project Splatter each month between 2013 and 2016 are similar, with peaks in relative mortality in March and October and a clear trough each year between June and August (figure 2). The mean proportion of roadkill pheasants rose across the 4 years from 0.35 (0.32–0.39 CI) in 2013 to 0.41 (0.38–0.44 CI) in 2016 (year: *χ*^2^ = 7.98, d.f. = 3, *p* = 0.046). There were some inter-annual variations, most notably a lower proportion of roadkill pheasants during the winter and early spring of 2013 compared to subsequent years (year × month: *χ*^2^ = 150.39, d.f. = 33, *p* < 0.001).

### 3.3. Differences in patterns of mortality before and after the introduction of large-scale pheasant releases

Prior to the introduction of large-scale pheasant releases (1957–1961), there was no discernible peak in mortality in any one month when the reports of roadkill pheasants were corrected for total reports of birds killed on the road (figure 1*a*). When the proportion of pheasants killed per month was considered, there was an approximately fivefold increase in relative mortality during June–August compared to levels observed during the rest of the year (figure 1*b*). By contrast, in 2013–2016 when large-scale releases were common, there was a distinct trough in mortality between June and August, being at least three times lower than at any other month of the year, when the reports of pheasant mortality were corrected for total reports of bird mortality (figure 1*a*). There was also a peak in February–April of approximately 12 times that seen during June–August and a smaller peak in September–November of approximately three times that seen during June–August, when the proportion of pheasants killed per month was considered (figure 1*b*).

The pattern of pheasant roadkill in the 1960s is similar to that of the next most commonly reported (group of) species in Project Splatter, pigeons. Pigeons also have a peak in the proportion of their annual total killed per month during their breeding season in June–August, and this pattern was similar in both the 1960s and 2010s (figure 1*b*). By contrast, the pattern for pheasant roadkill in the 2010s is one of a trough during this summer period with peaks in spring and autumn (figure 1*b*).

### 3.4. Differences in patterns of pheasant mortality across England

**Figure 3.**
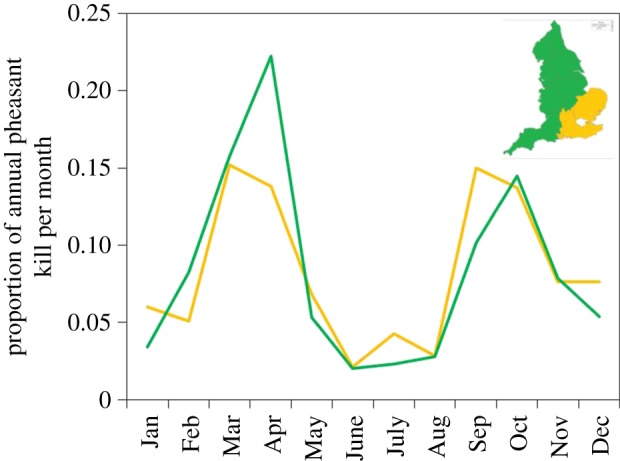
The proportion of all pheasants reported killed in vehicle collisions per year (2013–2016) in each month in regions of England with high (yellow line) and low (green line) levels of arable farming.

The proportions of roadkill pheasants did not differ between regions with high levels of arable agriculture and the rest of England (region: *χ*^2^ = 3.32, d.f. = 1, *p* = 0.069). However, there was a difference in the distribution of mortality each month between the two regions (region × month: *χ*^2^ = 78.16, d.f. = 11, *p* < 0.001) with the peak in mortality in non-arable regions in April being 61% higher than that of arable regions (figure 3).

### 3.5. Differences in patterns of mortality between the sexes

**Figure 4.**
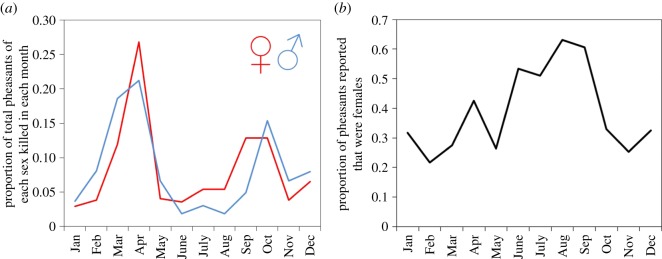
(*a*) The proportion of all pheasants reported killed in vehicle collisions per year (2013–2016) in each month that were females (red line) and males (blue line). (*b*) The proportion of pheasants reported killed by vehicle collisions in each month that were females.

Almost 70% more males (*n* = 753) than females (*n* = 444) were reported killed on roads. The time of year when they were reported also differed between the sexes (sex × month: *χ*^2^ = 69.7, d.f. = 11, *p* < 0.001) with roadkill of males being disproportionately reported earlier in spring and later in autumn compared to females (figure 4*a*). The proportion of roadkill pheasants that were females was highest during June–September (figure 4*b*).

## 4. Discussion

Pheasants, both prior to and after a substantial increase in population size due to intensive rearing and release, have consistently been reported as being killed in vehicle collisions around 12–13 times more than expected given their breeding population. This high proportion may be an artefact, arising because they are large birds and hence less agile, more conspicuous and resistant to decay and less likely to be removed by scavengers than smaller birds. When their biomass is accounted for, they were reported as killed on roads around 1.3–1.6 times more than expected. By contrast, pigeons, which are also reasonably large (approx. 500 g), were killed by vehicles between 0.6 and 2.3 times more than expected given their breeding population, and at levels about 88% of those expected given their biomass. Therefore, even when controlling for contributory factors related to size, pheasants were around twice as likely to be reported as roadkill as pigeons, suggesting some aspect of pheasant behaviour or ecology makes them unusually susceptible, rather than them simply being conspicuous or long-lasting. Their consistently high contribution to roadkill over the last 50 years may be because they are unusually susceptible to vehicle collisions because they are predominantly terrestrial, or, like other birds that are commonly killed by vehicles, are omnivorous and have short flight distances. They may also be commonly reported because they (especially males) are easily identifiable and widely recognized.

Despite their general and persistent susceptibility to death on roads, there has been a distinct change in the pattern of when deaths occur in the UK which corresponds to the introduction of large-scale releases and intensive game management, as well as a change in farming practice. By contrast, pigeons, the next most common (morpho)species reported killed on roads, showed no such change in their pattern of mortality since the 1960s with a consistent peak corresponding to their breeding period in both the 1960s and 2010s (figure 1*b*). In the 1950s and 1960s, prior to large-scale releases, the bulk of pheasant deaths were recorded during their breeding season between June and August (figure 1*a*). The peak during the breeding season prior to the 1960s may be because the verges of roads, if poorly tended, can provide an excellent nesting environment for pheasants and thus increase their exposure to traffic threat [12], or because road verges serve as linear features that may be especially attractive to males who typically establish territories along linear features such as woodland edges, hedges or ditches [8]. There may also be increased susceptibility during the breeding season because adults are engaging in more or riskier foraging to meet their own energetic requirements or supply their young [8]. By contrast, during the 2010s, when most pheasants are bred in captivity and releases are massive and widespread, the breeding season was the time when the fewest pheasant deaths were recorded. Instead, in this period, the bulk of pheasant deaths were recorded prior to the start of breeding between February and April with a second peak between September and November.

The autumn mortality peak seen in the 2010s survey data (figure 1*b*) may be a result of artificially reared birds dispersing from the pens where they were released in August. Such naive birds may be exploring the novel environment in the absence of parental guidance and hence be especially susceptible to death in a novel scenario (traffic) for which they have no evolutionary preparation or prior experience. That the peak may be due to dispersal is supported by the differences in mortality patterns between the sexes, with female peak mortality in autumn occurring a month (September) before that of males (October; figure 4*a*). Female pheasants tend to disperse earlier, further from and more directly away from release pens [32,33], rendering them susceptible to vehicle collisions earlier on. A peak is not seen at this time in the UK data prior to the 1960s, perhaps because birds in these surveys were predominantly wild-born and would have typically spent the first few weeks or months of life with their parents that may have led them to foraging sites away from road dangers, or warned juveniles of traffic threat.

We can conceive two explanations for the late winter/early spring peak in the 2010 data. It occurs at the time when males start to establish and defend territories and when overwinter supplementary feed has been withdrawn, marking the beginning of the ‘hungry gap’ [8]. Therefore, a peak in mortality may be because males are either prospecting for vacant or contestable territories or focused on territorial defence and thus are more exposed to traffic threat or less averse to such threat.

If the mortality peak is driven by territoriality, then we would expect that most pheasants killed at this time would be males seeking or defending territories. We would also have expected to have seen a similar peak in the pre-1960s populations because we do not expect that the breeding behaviour of birds has changed in just 50 years. We do not have data on the sexes of birds killed in the 1960s but for a subset of the 2010s data in which sex was reported, there was almost twice the proportion of males killed in February and March compared to females, but around 25% higher proportion of females killed in April compared with males (figure 4*a*). This gross difference in the reporting of sexes killed may partly be due to the conspicuous plumage of male pheasants being easier to detect and identify. However, although females may be cryptic in natural woodland, both sexes stand out against a tarmac background. Detailed observations of the behaviour and space use of male pheasants during the breeding season in the vicinity of roads would permit a better understanding of this hypothesis. Alternatively, if the mortality peak is driven by pheasants being forced to leave established feeding sites and search out new food supplies, then we would expect that in areas of the UK holding greater supplies of natural food, the mortality peak would be lower. The proportion of pheasants killed in a month was lower during this February–April period in areas of England (East and East Midlands) with high levels of arable farming (figure 3). Pheasants in such arable areas are more likely to find gleanings of seeds or fresh shoots from winter cereal crops to eat. We predict that if roadkill is driven by hunger, then pheasants killed on the road will be lighter or have less food in their crops than pheasants sampled away from roads in the same region.

Consequently, we suspect that both territoriality and a search for new food supplies may contribute to mortality in the spring. However, given that territorial behaviour is unlikely to have changed since the 1960s, we believe that the emergence of the spring peak in roadkill mortality is better explained by changes in game management with the current reliance on establishing high dependence on artificial food supplies which are withdrawn at the end of the shooting season, coupled with changes in agricultural practice. A finer-scale study recording roadkill levels in the region of estates that cease or continue spring feeding would permit an explicit test of this hypothesis.

Perhaps pheasants could have undergone evolutionary change in response to roadkill risk over the 50 years. Cliff swallows collected as roadkill exhibited an increase in wing length over a 30 year period compared with the population at large, and relatively fewer cliff swallows were killed on roads over this time [34]. This suggests that in a short time, morphology and perhaps corresponding behaviour may have been strongly selected by roadkill mortality. We have no evidence that such selection has acted on pheasant morphology or behaviour but we cannot exclude this possible explanation.

These changes in mortality patterns are instead likely (at least partially) due to changes in game management, perhaps in conjunction with changes in agricultural practice in the UK. However, this is unlikely to be simply a consequence of the increased scale of release. Released pheasants remain about 12 times more likely to be reported killed on a road as other birds over the past 50 years, even though numbers released have increased substantially. Therefore, this mortality appears to be an intrinsic property of pheasants, with their ecology, morphology and/or behaviour making them more likely than other species to be killed on roads. Crucially, over the 50 years the natal origin of pheasants has changed, from the majority encountered in the UK being wild-born to the majority being artificially reared. Artificial rearing produces many young birds that have been reared in the absence of adults and thus denied opportunities for learning. Consequently, human game management techniques must be deployed to ameliorate roadkill risks for released pheasants. If, as we observed, pheasants are more likely to encounter roads, and be killed on them, when they first leave their release pens then greater efforts to encourage them away from roads could be made over this relatively short time. Such efforts could include using dogging in (patrolling the shooting area on a daily basis with a dog in order to herd dispersed birds back towards a release pen) on routes between the pen and roads, or placing feeders in the opposite direction to roads from the release pen, or disruption of linear features such as hedges, ditches or streams that birds may follow from their release pen to nearby roads. If, as we observed, pheasants are more likely to encounter roads prior to the breeding season then continued game management should persist after the shooting season has finished. Changes in agricultural practice with removal of overwinter stubbles, hedges and rough ground has depleted natural overwinter food reserves. Therefore, artificial food provision could be maintained over the ‘hungry gap’ (February–May), ideally at sites to draw hungry birds away from roads. We have suggested some potential measures that we believe may ameliorate levels of roadkill and attendant risk to motorists, but emphasize that they need formal testing to determine their efficacy.

## Supplementary Material

Raw data used for analysis of 2010s roadkill patterns

## Data accessibility

Our novel raw data are available in the electronic supplementary material. We reference existing published sources of data that we used.
